# Degenerative Changes in the Disc, Facet Joint, and Paraspinal Muscle After Adolescent Idiopathic Scoliosis Surgery: A Five-Year MRI-Based Follow-Up

**DOI:** 10.7759/cureus.88116

**Published:** 2025-07-16

**Authors:** Takaaki Uto, Noriaki Yokogawa, Satoshi Kato, Takaki Shimizu, Satoshi Nagatani, Masafumi Kawai, Yuji Ishino, Kazuhiro Nanpo, Satoru Demura

**Affiliations:** 1 Department of Orthopaedic Surgery, Graduate School of Medical Sciences, Kanazawa University, Kanazawa, JPN

**Keywords:** adjacent segment degeneration, adolescent idiopathic scoliosis (ais), facet joint degeneration, ivdd (intervertebral disc degeneration), long-term follow-up after orthopedic surgery, lowest instrumented vertebra, mri evaluation, paraspinal muscle atrophy, spinal fusion surgery, srs-22

## Abstract

Background and objectives

This study aimed to comprehensively assess degenerative changes in the intervertebral disc, facet joint, and paraspinal muscle after adolescent idiopathic scoliosis surgery and to evaluate clinical outcomes at a five-year follow-up using magnetic resonance imaging (MRI). We hypothesized that the pattern of degeneration would differ based on the distal level of fusion.

Materials and methods

We retrospectively reviewed patients with adolescent idiopathic scoliosis who underwent surgery between 2006 and 2015. The patients were divided into two groups based on the lowest instrumented vertebra (LIV): a proximal LIV group (L2 or above) and a distal LIV group (L3 or below). Degeneration was evaluated using MRI with the Pfirrmann and Fujiwara scoring systems. Fatty infiltration of paraspinal muscles was specifically assessed at the L5/S1 disc level, distal to the surgical field, using the Goutallier scoring system. Clinical outcomes were measured using the Scoliosis Research Society-22 (SRS-22) Patient Outcome Questionnaire. Statistical comparisons were performed using the Mann-Whitney U test and Wilcoxon signed-rank test.

Results

The proximal LIV group comprised 21 patients, while the distal LIV group had 19 patients. Postoperatively, six of 40 patients (15%) exhibited progression of disc degeneration in mobile segments, with no significant difference between groups. The proximal LIV group showed significant progression of facet joint degeneration up to three levels below the fusion, whereas the distal LIV group showed similar changes up to two levels. However, the distal LIV group demonstrated greater overall facet degeneration. Paraspinal muscle degeneration at the L5/S1 level was observed in 19 of 40 patients (47.5%), with a higher prevalence in the distal LIV group (73.7% vs. 23.8%). Clinical outcomes were satisfactory and did not differ significantly between the groups.

Conclusions

At the five-year mark, this study suggests an association between a more proximal LIV (L2 or above) and less facet joint and paraspinal muscle degeneration in patients undergoing fusion for adolescent idiopathic scoliosis. However, these preliminary radiological findings did not correlate with clinical symptoms, which remained satisfactory in both groups. These results should be interpreted with caution, and extended follow-up is necessary to determine the long-term clinical significance of these degenerative changes.

## Introduction

Scoliosis is a complex spinal deformity characterized by the lateral curvature and rotation of the vertebrae. The causes of scoliosis are diverse and are broadly classified into several categories: congenital, neuromuscular, syndrome-related, idiopathic, and spinal curvature due to secondary reasons. Idiopathic scoliosis is the most common type encountered in general practice.

Untreated moderate-to-severe scoliosis can progress and lead to noticeable changes in appearance, increased back pain, and, in rare cases, can affect the lungs and heart, making it difficult to breathe and for the heart to pump. One of the main surgical approaches involves fusion of the vertebrae, rendering a portion of the spine solid. However, this fusion may increase the stress on non-fused segments, potentially accelerating their degeneration, which is a matter of concern for clinicians [1,2].

Historically, studies on Harrington and Cotrel-Dubousset instrumentation have examined the implications of choosing specific fusion levels for spinal mobility and postoperative pain [1]. In contrast, recent studies on pedicle screw instrumentation systems have investigated the effect of adding distal fusions on spinal mobility [2].

Several studies have specifically targeted degenerative changes in lumbar segments after long fusions. Investigations with a radiographic focus on adolescent idiopathic scoliosis (AIS) have predominantly focused on post-surgical disc degeneration in the lumbar segments [3-5]. Conversely, investigations employing modern instrumentation systems have used magnetic resonance imaging (MRI) to detect degenerative changes in mobile segments [6-8]. Expanding on this, some researchers have integrated facet joint degeneration into their evaluations [9], whereas others have focused on paraspinal muscle degeneration [10,11].

Biomechanically, fusion amplifies the stress on adjacent mobile segments, potentially hastening their degeneration [12]. A healthy lumbar disc distributes load evenly; however, the unique spatial orientation of the facets introduces complex motions that intertwine flexion-extension with both axial and lateral bending [13]. Research limited solely to one aspect, such as disc degeneration, offers an incomplete picture of the degenerative cascade in unfused segments.

Comprehensive MRI studies that evaluate discs, facets, and muscles together are rare. Our study aimed to investigate the progression of degenerative changes in non-fused segments of patients who have undergone surgery for AIS, particularly focusing on the sequence of degeneration between spinal components. While traditional perspectives have emphasized disc degeneration as the primary initiating factor [14,15], our study explores the possibility that degenerative processes might also commence in posterior spinal elements such as facet joints and paraspinal muscles. We hypothesize that in AIS surgery, these posterior components may also show early signs of degeneration, possibly influenced by altered biomechanics post-surgery. Furthermore, we hypothesize that the pattern of degeneration in these non-fused, posterior components may differ based on the distal level of fusion, potentially impacting long-term clinical outcomes. Therefore, the primary aims of this study were to (1) comprehensively evaluate degenerative changes in the disc, facet joints, and paraspinal muscles after AIS surgery and (2) compare these changes between patients with proximal versus distal lowest instrumented vertebra (LIV) levels.

This article was previously presented as a poster at the 2024 American Academy of Orthopaedic Surgeons (AAOS) Annual Meeting on February 12-16, 2024.

## Materials and methods

Patient population

This study was conducted in accordance with the tenets of the Declaration of Helsinki and approved by the Kanazawa University Ethical Committee of Clinical Research (IRB number: 2015-075). We retrospectively analyzed all scoliotic surgeries performed at our institution between 2006 and 2015. Of the 128 patients who underwent this procedure, 61 were diagnosed with idiopathic scoliosis. MRI data were collected and analyzed. Of these, 40 patients met the following inclusion criteria and were included in this study.

For the inclusion criteria, patients had to have been diagnosed with idiopathic scoliosis and undergone surgery for a progressive curve. They needed to be above 10 years of age, irrespective of sex. It was essential that they had undergone a preoperative MRI prior to their correction and fusion surgery and that their LIV was between T12 and L4. In addition, they had to have a minimum of a five-year follow-up and were evaluated with a postoperative lumbar MRI at the five-year mark.

Regarding exclusion criteria, we excluded patients with non-idiopathic scoliosis, such as congenital, neuromuscular, syndromic, or early onset types. Additionally, those with a history of previous spine surgery, revision surgery, neurological deficits, or less than five years of follow-up were also excluded from the study.

Outcome measures

All MRI scans were performed using a 1.5-Tesla scanner. Standard institutional lumbar spine protocols including sagittal and axial T2-weighted sequences were used for evaluation. Forty patients were divided into two groups to compare outcomes based on the distal extent of the fusion. The proximal LIV group was defined as having the LIV at L2 or higher (n = 21), while the distal LIV group was defined as having an LIV at L3 or lower (n = 19), following the classification by Burton et al. [16]. Lumbar MRIs, both preoperatively and at the five-year postoperative follow-up, were analyzed for disc and facet joint degeneration using the Pfirrmann and Fujiwara scoring systems, respectively [14,17].

**Figure 1:**
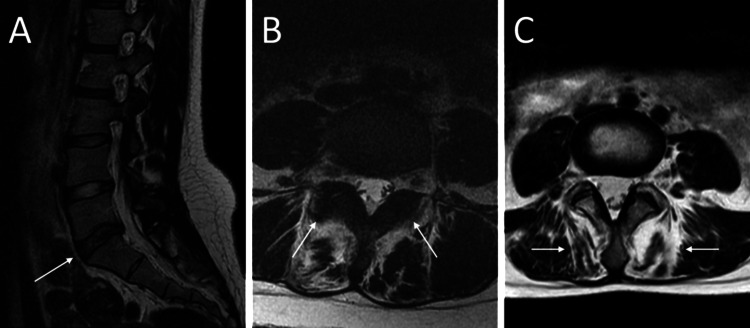
Degenerative changes observed on MRI. (A) Sagittal T2-weighted image showing disc degeneration at the L5-S1 level, characterized by an inhomogeneous signal and indistinct separation between the nucleus and annulus (white arrow), rated as Pfirrmann Grade 3. (B) Axial T2-weighted image demonstrating facet joint degeneration with pronounced osteophytes and severe osteoarthritis (white arrows), rated as Fujiwara Grade 4. (C) Axial T2-weighted image showing paraspinal muscle degeneration at the L5/S1 level with over 50% fatty infiltration and muscle atrophy (white arrows), assessed as Goutallier Grade 4.

To differentiate between degenerative changes resulting from altered biomechanics and those caused by direct surgical trauma, we specifically assessed fatty infiltration of the multifidus and erector spinae muscles at the L5/S1 disc level. This level was chosen because it was distal to all fusion constructs in our cohort, thereby minimizing the confounding influence of iatrogenic muscle injury from the surgical approach. This evaluation was performed using the Goutallier scoring system [18,19]. A summary of these grading systems is provided in Table 1. A visual representation of these degenerative changes is shown in Figure 1. Clinical outcomes were assessed preoperatively and five years postoperatively using the Scoliosis Research Society-22 (SRS-22) Patient Outcome Questionnaire [20].

**Table 1:** Stages and brief description of each scoring system.

Pfirrmann scoring system	
Grade Ⅰ	The disc appears normal with a bright white color (indicative of high-water content) and a clear distinction between the nucleus and annulus.
Grade Ⅱ	The disc has a slightly inhomogeneous structure with a normal or slightly decreased intensity. The nucleus and annulus are still distinguishable.
Grade Ⅲ	The disc shows an inhomogeneous structure with intermediate signal intensity. There is an unclear distinction between the nucleus and annulus.
Grade Ⅳ	The disc displays an inhomogeneous structure with low signal intensity, and the nucleus and annulus are not distinguishable. The disc space may be slightly reduced.
Grade Ⅴ	The disc has a collapsed disc space with very low signal intensity and no distinction between the nucleus and annulus.
Fujiwara scoring system	
Grade 1	Normal: No visible signs of degeneration
Grade 2	Mild degeneration: characterized by slight joint space narrowing or the presence of mild osteophytes
Grade 3	Moderate degeneration: Indicated by moderate osteophytes and/or sclerosis of the joint
Grade 4	Severe degeneration: Defined by large osteophytes and significant sclerosis, showing advanced degenerative changes
Goutallier scoring system	
Grade 0	No fatty infiltration
Grade 1	Some fatty streaks
Grade 2	Less fat than muscle
Grade 3	Equal amounts of fat and muscle
Grade 4	More fat than muscle

Statistical analysis

Statistical analyses were conducted using IBM SPSS Statistics for Windows, Version 26.0 (Released 2018; IBM Corp., Armonk, New York, USA). The significance level was set at p < 0.05. To ensure the normal distribution of data, the Shapiro-Wilk test was used. Two types of tests were used to evaluate participant demographics, depending on the normality of the data: the independent t-test was applied for normally distributed data, while the Mann-Whitney U test was used for data that did not follow a normal distribution. Wilcoxon tests were employed to assess changes in degeneration before and after surgery. Additionally, the Mann-Whitney U test was used to compare postoperative degenerative differences between the proximal and distal LIV groups and to compare clinical outcomes between these two groups.

To minimize assessment bias, all radiological evaluations were performed by a single, experienced orthopedic surgeon who was blinded to the patient's clinical information and surgical group allocation (proximal vs. distal LIV). A critical aspect of the study was to ensure consistency and reliability in evaluations, which were carried out by a single individual. To assess the reliability of this evaluator, the intra-rater reliability was calculated for all 40 patients. The intraclass correlation coefficient (ICC) was found to be 0.794, with a 95% confidence interval of 0.764-0.819. This ICC value was considered "good," indicating a high level of consistency in the evaluator's ratings across different assessments.

## Results

Patient characteristics

In this study, including 40 patients who underwent scoliosis surgery, patients were divided into two groups based on the LIV level. The proximal LIV group included 21 patients, and the distal LIV group included 19 patients. Upon a detailed analysis of patient demographics, no statistically significant differences were found between these groups. This similarity was evident in various demographic parameters, including BMI. The average BMI across all patients was 19.47 ± 2.09 kg/m², with both the median and mode being 19 kg/m², indicating a relatively uniform distribution of body weight.

Regarding the extent of the fusion, the average number of spinal levels fused was 9.125 ± 2.80, with the median and mode being nine levels. This uniformity in the extent of fusion across patients provides a homogenous basis for comparing post-surgical outcomes. The demographic and surgical characteristics are summarized in Table 2.

**Table 2:** Demographic and surgical characteristics of the study cohort. LIV: lowest instrumented vertebra.

	All patients (n=40), mean ± SD	Proximal LIV group (n=21), mean ± SD	Distal LIV group (n=19), mean ± SD	p-value
Age at surgery (years)	16.6 ± 3.7	16.2 ± 2.8	17.0 ± 4.6	0.989
Female (n)	37	19	18	0.614
Height (cm)	154.1 ± 5.9	154.1 ± 5.7	154.0 ± 6.3	0.919
Weight (kg)	46.2 ± 6.4	46.8 ± 5.5	45.5 ± 7.3	0.548
BMI (kg/m^2^)	19.5 ± 2.1	19.8 ± 2.2	19.2 ± 2.0	0.370
Operative time (min)	405 ± 134	423 ± 120	387 ± 147	0.415
Blood loss (mL)	682 ± 490	750 ± 468	615 ± 515	0.296

The distribution of the LIV across the patients varied, with seven patients having their LIV at T12, eight at L1, six at L2, 17 at L3, and two at L4. This distribution was reflective of the diverse nature of scoliosis cases and the tailored approach to surgical intervention.

Disc degeneration

In the analysis of 40 patients, MRI scans revealed that six patients (15%) showed signs of disc degeneration progression. This degeneration was predominantly observed at the lower lumbar levels, specifically at L3-L4, L4-L5, and L5-S1. Over the five-year follow-up period, our statistical analysis indicated that there were no significant changes in the Pfirrmann scores from pre- to post-surgery. The consistency of the Pfirrmann scores over time was also reflected in the comparison between the two surgical groups. When we examined the postoperative MRIs, the Pfirrmann scores did not show any significant differences between the proximal LIV and distal LIV groups.

Facet degeneration

**Figure 2:**
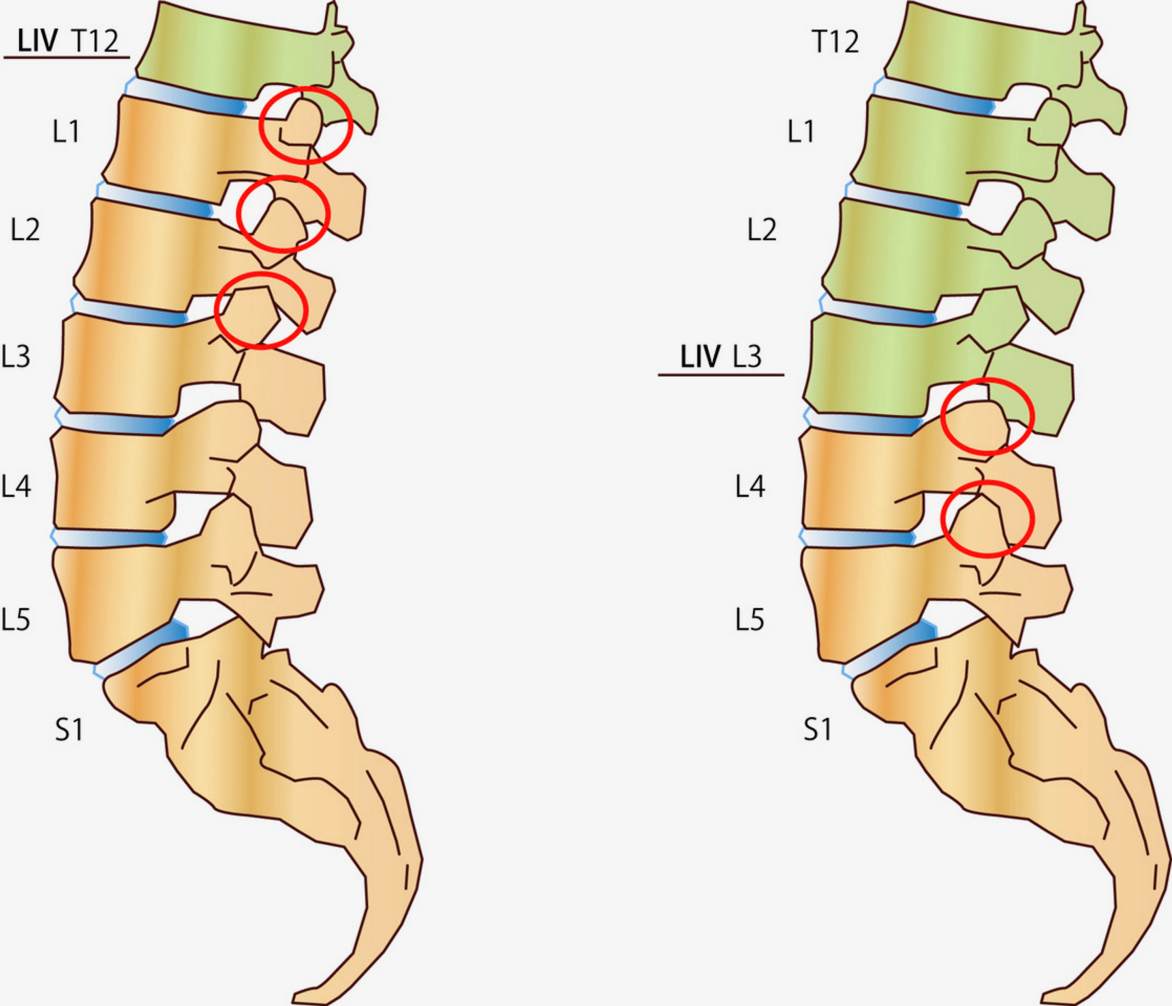
Schematic representation of facet degeneration patterns. This figure illustrates the different spatial patterns of facet joint degeneration observed at the five-year follow-up. In the proximal LIV group (fusion ending at L2 or above), significant degeneration was noted to extend up to three levels below the fusion construct. In contrast, in the distal LIV group (fusion ending at L3 or below), degeneration was primarily observed up to two levels below the fusion. LIV: lowest instrumented vertebra.

Six of the 40 patients displayed no facet joint degenerative changes at any level during the five-year follow-up period. Facet joints showed continuous degeneration not only between adjacent vertebrae but also distally, as detailed in Table 3. In the distal LIV group, postoperative Fujiwara scores were more increased at the L4/5 level compared to those observed in the proximal LIV group. A schematic representation of these different degenerative patterns is shown in Figure 2. A comparison of degenerative changes is presented in Table 4.

**Table 3:** Incidence of facet joint degeneration progression by LIV and spinal level. Values represent the number of patients (%) showing an increase of at least one Fujiwara grade at the five-year follow-up. LIV: lowest instrumented vertebra.

	Facet joint degeneration progression at each level					
LIV	T12-L1	L1-L2	L2-L3	L3-L4	L4-L5	L5-S1
T12 (n=7)	5 (71%)	5 (71%)	2 (29%)	0 (0%)	0 (0%)	0 (0%)
L1 (n=8)	-	5 (63%)	4 (50%)	2 (25%)	2 (25%)	0 (0%)
L2 (n=6)	-	-	3 (50%)	1 (17%)	0 (0%)	0 (0%)
L3 (n=17)	-	-	-	7 (42%)	7 (42%)	1 (6%)
L4 (n=2)	-	-	-	-	1 (50%)	0 (0%)

**Table 4:** Comparison of preoperative and five-year postoperative degenerative scores. Values are presented as mean ± standard deviation. The p-value represents the comparison of the difference (five-year follow-up minus preoperative score) between the proximal and distal LIV groups. Disc degeneration was assessed by Pfirrmann grade, facet degeneration by Fujiwara score, and fatty infiltration by Goutallier score. LIV: lowest instrumented vertebra. ^*^Statistical significance at p < 0.05.

Degenerative change	Spinal level	Proximal LIV group (n=21)			Distal LIV group (n=19)			p-value
		Preoperative	Five-year	Difference	Preoperative	Five-year	Difference	
Disc degeneration	L3-L4	2.0 ± 0.0	2.0 ± 0.0	0.0 ± 0.0	2.0 ± 0.0	2.1 ± 0.2	0.1 ± 0.2	0.769
	L4-L5	2.1 ± 0.4	2.1 ± 0.5	0.1 ± 0.2	2.1 ± 0.3	2.2 ± 0.5	0.1 ± 0.2	0.916
	L5-S1	2.1 ± 0.2	2.1 ± 0.4	0.1 ± 0.3	2.1 ± 0.2	2.1 ± 0.3	0.1 ± 0.2	0.643
Facet degeneration	L3-L4	1.2 ± 0.6	1.5 ± 0.8	0.3 ± 0.6	1.8 ± 0.8	2.4 ± 1.0	0.7 ± 0.9	0.232
	L4-L5	1.1 ± 0.2	1.1 ± 0.5	0.1 ± 0.3	1.3 ± 0.7	2.0 ± 1.1	0.6 ± 0.9	0.012^*^
	L5-S1	1.1 ± 0.2	1.1 ± 0.2	0.0 ± 0.0	1.2 ± 0.4	1.3 ± 0.6	0.1 ± 0.5	0.282
Fatty infiltration of paraspinal muscles	L5/S1	1.9 ± 0.4	2.1 ± 0.5	0.2 ± 0.4	2.1 ± 0.6	3.0 ± 0.7	0.9 ± 0.6	0.003^*^

Fatty infiltration of paraspinal muscles

Muscle degenerative changes at the L5/S1 level, distal to the fusion construct, were a notable observation. Nearly half of the patients, 19 out of 40 (47.5%), exhibited these changes. The prevalence of muscle degeneration differed between the two surgical groups. Among the 21 patients in the proximal LIV group, 5 (23.8%) showed muscle degenerative changes at L5/S1. In contrast, in the distal LIV group, a higher proportion of patients, 14 out of 19 (73.7%), exhibited such changes. To quantify these muscle changes, the Goutallier score was used. The scores were found to be elevated in both surgical groups, indicating noticeable muscle degeneration post-surgery.

Clinical outcomes

We examined clinical outcomes across all domains of the SRS-22 questionnaire. The data underscore a similarity in clinical outcomes between the two groups, as shown in Table 5. The average SRS-22 subtotal score across all patients was 4.24 ± 0.34, indicating overall satisfactory clinical outcomes. Additionally, neurological evaluation results across this cohort remained consistently normal throughout the follow-up period.

**Table 5:** Comparison of preoperative and five-year postoperative SRS-22 scores. Values are presented as mean ± standard deviation. The p-value represents the comparison between preoperative and five-year postoperative scores within each group. LIV: lowest instrumented vertebra; SRS-22: Scoliosis Research Society-22. ^*^Statistical significance at p < 0.05.

SRS-22 component	Proximal LIV group (n=21)			Distal LIV group (n=19)		
	Preoperative	Five-year	p-value	Preoperative	Five-year	p-value
Pain	4.40 ± 0.53	4.55 ± 0.35	0.883	4.47 ± 0.53	4.50 ± 0.44	0.851
Self-image	2.47 ± 0.43	3.75 ± 0.60	<0.001^*^	2.60 ± 0.43	3.77 ± 0.74	<0.001^*^
Function	4.60 ± 0.49	4.67 ± 0.25	0.613	4.51 ± 0.55	4.58 ± 0.34	0.681
Mental health	3.91 ± 0.49	4.41 ± 0.54	0.038^*^	3.91 ± 0.76	3.92 ± 0.97	0.986
Satisfaction	3.57 ± 0.70	4.11 ± 0.60	0.013^*^	3.05 ± 1.04	3.96 ± 0.51	0.009^*^
Subtotal	3.82 ± 0.39	4.32 ± 0.27	<0.001^*^	3.84 ± 0.44	4.15 ± 0.39	0.060

Furthermore, a Spearman's correlation analysis was performed to assess the relationship between postoperative degenerative scores and SRS-22 domain scores. The analysis revealed no significant correlation between the degree of any degenerative change and the corresponding clinical outcomes at the five-year follow-up (p > 0.05 for all correlations). There were no complaints related to mobility issues reported during the entire five-year span, suggesting the successful maintenance of functional mobility post-surgery.

## Discussion

The "three-joint complex" in the lumbar spine, consisting of the intervertebral disc and the two facet joints at each segment, plays a critical role in lumbar spine biomechanics [21]. The intervertebral disc, situated anteriorly, works with the posteriorly paired facet joints to carry spinal loads. In typical conditions, these facet joints are responsible for transmitting 3% to 25% of the segmental load. Understanding the mechanics and degenerative processes of this complex is crucial for addressing lumbar spine pathologies.

Degeneration within this complex often initiates in the intervertebral disc, leading to facet joint degeneration. However, this sequence can vary, as facet joint degeneration can independently occur and, at times, even precede disc degeneration. The design of the lumbar three-joint complex, which allows extensive flexion motion while preventing gross rotatory instability, is central to these processes.

Although there is general agreement that longer follow-up is needed to assess degenerative changes after AIS surgery, there has been insufficient research to determine at which sites degeneration begins early postoperatively. Our study addresses this gap by investigating the early onset of degeneration at specific sites after AIS surgery.

Our study, conducted over five years following idiopathic scoliosis surgery, demonstrates that fusion terminating at L2 or above leads to less degeneration in facet joints and reduced fatty infiltration in paraspinal muscles compared to fusion extending to L3 or below. This finding is vital for surgical planning, highlighting the need to carefully choose surgical strategies based on individual patient requirements.

Furthermore, it is critical to consider how surgical interventions impact muscle function and spinal biomechanics [22]. Changes in spinal structure and stability after surgery, especially with different distal fusion levels, can affect spinal biomechanics. These changes may result in an altered load and stress distribution across spinal segments, impacting adjacent muscles and joints. Surgical manipulation of posterior spinal elements during scoliosis correction can directly influence the health and function of paraspinal muscles. These factors could explain the observed variations in degeneration patterns. A deep understanding of these impacts is essential for developing tailored postoperative management strategies to optimize functional outcomes.

Overall, the demographic consistency between the proximal and distal LIV groups strengthens the validity of our comparative analysis, ensuring that any differences observed in surgical outcomes can be more confidently attributed to the surgical approach rather than underlying patient characteristics.

MRI has increasingly become the method of choice for obtaining detailed anatomical images, particularly for structures such as spinal discs, facet joints, and muscles [10,11]. The key advantages of MRI are its non-invasive nature and lack of ionizing radiation, making it a safe imaging option. These advantages are significant in clinical settings where patient safety is a paramount concern.

Despite the general preference for MRI in assessing disc degeneration [14,23], its superiority over computed tomography (CT) scans in evaluating facet joint degeneration continues to be debated [24]. While MRI is widely regarded as the better option for disc assessments, both MRI and CT scans have demonstrated comparable effectiveness in depicting morphological changes in the facet joints [25]. However, in pediatric cases, MRI is generally preferred to minimize radiation exposure to young patients [14,26].

In our study, no significant differences in disc degeneration were observed among patients at the five-year postoperative follow-up. However, degenerative changes were noted in the distal mobile segments, especially in the lower lumbar region. These findings do not align with the results of previous studies, such as those by Nohara et al. [8], which suggested an increase in disc degeneration correlated with a more distal LIV at the 10-year evaluation. This discrepancy may indicate that, regardless of the LIV position, disc degeneration in the lower lumbar region can emerge initially.

Our study also highlighted facet degeneration, which was evident up to three levels below the fusion mass. This finding does not align with those of Enercan et al., who observed increased facet joint degeneration at levels immediately below the LIV [9]. Interestingly, we found facet degeneration to be more pronounced in the proximal LIV group, extending up to three levels below the fusion mass. In contrast, the distal LIV group showed significant degeneration up to two levels below the fusion mass, suggesting a variation in degenerative patterns based on the surgical approach.

Disc degeneration typically originates from the most distal mobile segment, while facet degeneration often begins immediately below the fusion mass [8,9]. This pattern contrasts with findings from other research, such as Dehnokhalaji et al., which identified the highest degenerative changes at the L2-L3 level [23]. Our study contributes to this body of knowledge by providing additional insights into the patterns of post-surgical degeneration.

Furthermore, our study examined the fatty infiltration of paraspinal muscles at the L5/S1 level, an area that is remote from the direct surgical approach, which has seen limited research. By focusing on a level distal to the fusion instrumentation, we aimed to minimize the confounding effects of direct surgical trauma and instead capture the secondary effects of altered spinal biomechanics. We observed that lumbar spinal degeneration appears to begin in the posterior structures of the spine, contrary to the common belief that it originates from anterior spinal structures [14,15]. While disc degeneration is often linked to the onset of osteoarthritis in the lumbar facets, our findings, along with studies like that of Lewin [27], suggest that it is not the sole trigger. Additionally, gender-based differences in motion segments and the complex interplay between disc and facet joint degeneration on spinal flexibility might also influence these degenerative patterns [15,28].

A key finding of our study was the confirmed lack of correlation between the degenerative changes observed on MRI and the clinical outcomes reported by patients via the SRS-22 questionnaire. Our formal correlation analysis showed that even in cases with more advanced radiological degeneration, patients did not report worse pain, function, or self-image at the five-year mark. This dissociation is clinically important, as it suggests that asymptomatic degenerative changes are common in this young population in the medium term and mirrors findings from other research [13]. It underscores the complexity of postoperative outcomes, where factors other than structural degeneration, such as psychosocial factors or neuromuscular adaptation, likely play a significant role in a patient's perceived well-being.

Our study is original as it integrates the evaluation of three key degenerative changes (discs, facet joints, and muscles) post-scoliosis surgery, which is not commonly addressed in existing literature. We believe that this comprehensive approach provides a more complete understanding of the impact of scoliosis surgery on spinal health.

Another aspect not covered in this study is the potential influence of postoperative rehabilitation. We did not collect standardized data on physiotherapy protocols, which may play a role in mitigating muscle atrophy and influencing clinical symptoms. The impact of tailored physical therapy on the progression of these degenerative changes and on patient-reported outcomes remains an important question for future prospective investigations.

Limitations

This study has several important limitations that must be acknowledged. First, the retrospective design and the small sample size (n = 40) limit the statistical power of our analyses and the generalizability of our findings. The small subgroups increase the risk of Type II errors, where a true difference may have been missed. Second, our study did not perform a multivariate analysis to account for potential confounding variables, such as preoperative curve severity, individual physical activity levels, or baseline muscle condition. A meaningful multivariate analysis was not statistically feasible with the current sample size. Third, while all radiological assessments were performed by a single blinded assessor to ensure consistency (intra-rater ICC = 0.794), inter-rater reliability was not assessed. Additionally, specific MRI acquisition parameters were not uniform across the long study period, although all scans were performed on a 1.5T system. Finally, the five-year follow-up period may not be sufficient to capture the long-term symptomatic impact of the observed degenerative changes. Therefore, our findings should be interpreted as preliminary, highlighting the need for larger, multicenter prospective studies to confirm these results.

## Conclusions

Our five-year follow-up suggests that postoperative degeneration after idiopathic scoliosis surgery may primarily affect the posterior spinal elements. We observed an association where fusion terminating at L2 or above was linked to less facet joint and paraspinal muscle degeneration compared to fusion with a more distal LIV. While these preliminary findings hint that preserving more mobile lumbar segments could mitigate biomechanical stress, this study was not designed to establish causality. Crucially, these distinct degenerative patterns observed on MRI did not correlate with clinical symptoms at the five-year mark, as clinical outcomes were satisfactory in both groups. This lack of correlation is a key finding of our study. Therefore, our results should be interpreted with caution. Further long-term, prospective studies are essential to validate these initial observations and to determine if and when these asymptomatic radiological changes become clinically significant.
